# Polish Version of the Resilience Scale (RS-14): A Validity and Reliability Study in Three Samples

**DOI:** 10.3389/fpsyg.2018.02762

**Published:** 2019-01-17

**Authors:** Janusz Surzykiewicz, Karol Konaszewski, Gail Wagnild

**Affiliations:** ^1^Faculty of Philosophy and Education, Katholische Universität Eichstätt-Ingolstadt, Eichstätt, Germany; ^2^Faculty of Education, Cardinal Stefan Wyszynski University in Warsaw, Warsaw, Poland; ^3^Faculty of Pedagogy and Psychology, University of Białystok, Białystok, Poland; ^4^The Resilience Center, Billings, MT, United States

**Keywords:** resilience, youth, measuring resilience, early adulthood, adolescent, juveniles

## Abstract

There has been a need for an instrument which not only can adequately evaluate trait-like resilience, but also can be applied to Polish adolescents and young adults. The purpose of this study was to validate the Resilience Scale RS-14 (Wagnild, 2009a,b). We aimed to examine and assess the psychometric properties of the Polish version in three different samples. The first sample was made up of adolescents aged 13–17 (*N* = 400).The second sample was made up of a problem group aged 13–18 (*N* = 656) who had special needs and attended either Probation Centers, Youth Sociotherapy Centers, or Youth Educational Centers. The third sample was made up of students in early adulthood aged 19–27 (*N* = 1,659). Exploratory and confirmatory factor-analytic methods were employed. CFA demonstrated a good fit of the factor structure in all three samples. The original one-dimensional structure of the RS short form was confirmed. All items loaded (>0.40) onto 1 factor, indicating cohesive structure for a 1-factor model explaining 35.02% of the variance in the whole sample, 34.62% in the young adolescent sample, 31.11% in the problem sample, and 38.51% in the early adulthood sample. Descriptive statistics, reliability (young adolescence α = 0.85, problem sampleα = 0.82 early adulthood α = 0.87) and validity data were calculated; test-retest showed good stability [*r*_(40)_ = 0.88; *p* < 0.001]. The validity of the scale RS-14 was applied in two groups (the *N* = 382 early adulthood aged 19–27, and the *N* = 120 problem group aged 13–18) and was subsequently evaluated. The RS-14 correlated significantly, as expected, with measures of positive concepts (satisfaction with life). Results showed that resilience was negatively related with indexes of perceived stress and the dimension-of-depression. Findings support the RS-14 to be a valid and useful instrument for assessing resilience in diverse Polish adolescent groups, including those with special needs and those in early adulthood.

## Introduction

Theoretical assumptions and empirical findings show that resilience can be variously conceptualized either as an unidimensional or a multidimensional construct. The given heterogeneity in understanding the concept of resilience has emerged in the debate regarding the distinction between a person's boundaries (both internal and external) as stable protective factors and mechanisms. Personal characteristics, (understood either to be simply personal features or else to be stable personality traits), could be genetically transferred, could moderate the impact of negative stressors, support the ability for positive adaptation, facilitate the achievement of individuals' developmental tasks, and help individuals to achieve good adjustment and to cope with adversity and trauma. Conceptualization of resilience as a multidimensional construct arises from the understanding that resilience is a dynamic process of the interaction among different constitutional, biological, cognitive, interpersonal, and contextual factors. “Resilience is understood to be a dynamic, adaptive process that can start at any given moment in life and is usually represented as effectively mobilizing internal and external resources when a person is initially confronted with adverse life situations, events,” or even traumatic experiences—and when that person adapts, deals with, and recovers rapidly from such major adversities (Wagnild and Young, 1993; Lutha and Cicchetti, 2000; Luthar et al., 2000; Fergus and Zimmerman, 2005; Wright and Masten, 2005; Mancini and Bonanno, 2009; Feder et al., 2010; Gartland et al., 2011; Masten, 2011; Bonanno et al., 2012; Fletcher and Sarkar, 2012; Masten and Narayan, 2012).

“The interaction between the individual and the different contextual aspects leads to attitudes that elicit sustained positive outcomes with a continuous learning process of renewing and balancing situations” (Windle et al., 2011; Wiles et al., 2012; Wong and Wong, 2012; Donnellan et al., 2015). A typically “outcome-oriented approach regards resilience as a function or behavioral outcome that can conquer and help individuals to recover from adversity” (Harvey and Delfabbro, 2004; Masten, 2011; Rutter, 2012). There is a difference between resilience as “adaptive outcomes in the face of adversities and coping as a set of cognitive and behavioral strategies used by an individual to manage the demands of stressful situations” (Folkman and Moskowitz, 2004). While researching “resilience, it is necessary to be sensitive to the sociocultural factors that contextualize how it is defined by different populations” (Campbell-Sills and Stein, 2007; Ungar and Liebenberg, 2011; Wyche et al., 2011; Aiena et al., 2015).

## Resilience Research: Outcomes Among Youth

Those conceptual aspects of resilience, as they relate to the periods of youth and adolescence, “have gained particular attention in the search of such factors that may buffer the negative effects of adversities,” chronic stress and traumas that are especially significant in these stages of development. The resilience of youth “has been studied in a variety of aspects and contexts where young people face adversity. As an individual difference variable, trait resilience has been found to improve well-being and promote recovery from stressful situations” (Catalano et al., 2004, 2011; Taku, 2014; Ying et al., 2014; Oginska-Bulik and Kobylarczyk, 2016). “The more the adolescents experienced high levels of resilience, the more they felt themselves able to cope with novelty in various domains of life.” They tended to use almost all thinking styles (Sagone and De Caroli, 2013, 2014) and were better able to self-regulate (Veselska et al., 2009). The positive emotions of students “predicted increases in both resilience and life satisfaction” (Liu et al., 2013). Their “negative emotions had weak or null effects and did not interfere with the benefits of the positive emotions. Positive emotions also mediated the relation between baseline and final resilience, but life satisfaction did not” (Cohn et al., 2009, p. 361). Resilience is relevant to the life satisfaction of adolescents because “students with higher resilience levels had a better subjective quality of life and a better perception of the educational environment,” especially in scholastic context (Abolghasemi and Varaniyab, 2010; Tempski et al., 2015; Patry and Ford, 2016). Zuill (2016) explored whether individual resiliency factors influenced the academic success of foster care adolescents. In addition, “the more individuals reported high levels of resilience, the more they perceived themselves efficient” (Schwarzer and Warner, 2013).

“Resilience is a key indicator of an individual's successful adaptation to changes in life circumstances” (Diener et al., 1999; Abolghasemi and Varaniyab, 2010). It is also a supporting factor for adolescents to be invulnerable to adverse situations and “to be less likely to engage in risky behaviors” (Coleman and Hagell, 2007a,b; Earvolino-Ramirez, 2007; Gardner et al., 2008). Mowder et al. (2010) “found that ‘average’ resiliency was associated with less serious discipline problems” (Mowder et al., 2010). “Resilient youth exhibit fewer symptoms of anxiety and depression and a significantly reduced risk of suicidal behavior” (Sharaf et al., 2009; Salazar-Pousada et al., 2010; Hjemdal et al., 2011). “Resilient youth have been found to show greater resistance to negative peer influence associated with risky behaviors” (Rubin et al., 1998), and to “avoid psychosocial problems leading to addictive behavior” (Ali et al., 2010). Hall and Webster (Hall and Webster, 2007a,b) reported that resilience factors serve often “as buffers to life stressors and can serve as a protective mechanism for alcohol use, age at the onset of drinking, and affective factors.” The findings of the research have also shown that protective factors are important in understanding desistance from offending (Carr and Vandiver, 2001; Borum et al., 2002; Lodewijks et al., 2010; Fougere and Daffern, 2011). Mowder et al. (2010), when exploring the resiliency aspects and vulnerabilities of juvenile offenders, identified different cluster profiles of internal and external variables labeled to vulnerability and resilience, i.e., low resource vulnerability and average resiliency having influence on behavior. Not many studies have focused on the role of, nor characterized the measurement of resiliency aspects within youth and adolescents who have special needs, who are in foster care, who have externalized problem behavior or who, because of legal offenses, have come into contact with the Justice (Wright and Masten, 2005; Gibson, 2016; Gibson and Clarbour, 2017). In the above-mentioned aspects, it is therefore necessary not only to develop new tools, but also to validate existing tools.

## Measurement of Resilience Among Youth

As acknowledged by many researchers, the Resilience Scale 25 and Resilience Scale 14 (short version) have a long history of successful testing of validity. RS has been reported as one of the most appropriate instruments to measure resilience in the adolescent population. Ahern et al. (2006) “in their review of measures of resilience, assess six standardized measures, but point out especially the Resilience Scale as suitable for use with adolescents.” Windle et al. (2011) found that among 15 measurements only three were used specifically for adolescents and young adults (ages 12–23), including RS-25. Even if they did express critical opinion, they acknowledged that the RS-25 “measure appears to have had the widest application out of those identified and has been efficiently used with youth and adolescents” (Ahern et al., 2006; Windle et al., 2011).

As of today, available developed resilience scales do consist of a wide range of constructs which ascertain the extent to which at a given point of the life use can be made (Smith-Osborne and Whitehill Bolton, 2013; Artuch-Garde et al., 2017). However, “as a systematic review on resilience scales has revealed, most resilience measures assess the availability of protective factors that facilitate resistance to psychosocial dysfunction” (Windle et al., 2011). There are not many relevant, comprehensive measurement tools regarding young people (Lutha and Cicchetti, 2000; Windle et al., 2011; Aiena et al., 2015; Gibson, 2016). “Conceptual discrepancies hinder the evaluation and comparison of research findings, preclude meta-analysis, and make it difficult to operationalize the construct for measurement purposes” (Rutter, 1999, 2012; Ahern et al., 2006; Cohn et al., 2009; Mancini and Bonanno, 2009; Gartland et al., 2011; Windle et al., 2011; Bonanno et al., 2012; Liu et al., 2013; Smith-Osborne and Whitehill Bolton, 2013; Pangallo et al., 2015; Artuch-Garde et al., 2017). Any important development in the measurement of resilience has come from those scholars who have seen a need for the operationalization of resilience as personal aspects, “a trait-like conception, focused on a set of personal characteristics that mediate the effects of adverse life conditions on psychological adjustment” (Hjemdal, 2007; Castillo and Dias, 2009; Ungar and Liebenberg, 2009; Aiena et al., 2015).

“Some scales, such as Wagnild and Young's (1993) long version of Resilience Scale (RS-25) and short version RS-14 (Wagnild and Young, 1993; Wagnild, 2009b) and the Conner-Davidson Resilience (CD-RISC), represent such a construct option, based on cognitive/individual factors of resilience” (Burt and Paysnick, 2012). “They have been developed with adult populations yet have been used frequently with adolescents” (Jørgensen and Seedat, 2008; Pritzker and Minter, 2014). In the meantime, there has been expanded growing interest to develop new conceptual work (Wagnild, 2009a,b). Recently Gibson and Clarbour (2017) have proved validity of such measurement “in male adolescent offenders within the UK, using the Resiliency Scales for Children and Adolescents (RSCA)” (Prince-Embury, 2008; Prince-Embury and Courville, 2008). Scholars have suggested conducting further exploration of the construct validity, “in particular, in relation to young people's responses and reactions to incarceration” (Gibson and Clarbour, 2017).

Based on review of the literature, Pritzker and Minter (2014) identified six published validation studies with RS-14 among adolescents. (Nishi et al., 2010; Salazar-Pousada et al., 2010; Damásio et al., 2011; Kwon and Kwon, 2014; Pritzker and Minter, 2014; Aiena et al., 2015). Currently there are additionally new validations of RS-14 with adolescents (Castillo and Dias, 2009; Callegari et al., 2016; Madewell and Ponce-Garcia, 2016). Smith-Osborne and Whitehill Bolton (2013) also confirm that, while identifying among 11 scales measuring resilience with four specifically designed for application with youth, the one scale mostly used was RS-14 (Smith-Osborne and Whitehill Bolton, 2013). As indicated, researchers use RS-14 very often and our research fits into the mainstream of adaptation of scales for young people.

The Wagnild and Young (1993) “Resilience Scale” was developed “with the intention of measuring an individual level of resilience, understood as a relatively stable personal resource, being a positive personality trait that can be activated or used as personal competence and acceptance of self and life, all of which facilitates personal adaptation, i.e., coping with change or misfortune.” This concept incorporates the ability of an individual to recover from an adverse event by drawing upon internal and external sources of support, referring in this way to adaptive aspects of resilience. Wagnild and Young (1993) “originally suggested a five factor theoretical model, developed through qualitative analyses with a community sample of elderly women: equanimity (a balanced perspective of one's life), meaningfulness (the understanding that life is meaningful and valuable), perseverance (the ability to keep going, even after setbacks), self-reliance (the belief in one's abilities and awareness of limitations) and existential aloneness (the recognition of one's unique path and acceptance of one's life) (p. 167–168).” These components by exploratory analyses have been grouped in “two main factors: *personal competence* (e.g., self-reliance, independence, invincibility, mastery, resourcefulness, and perseverance) and *acceptance of self and life* (e.g., adaptability, flexibility, and balanced perspective of life)” (Wagnild, 2009a). However, factor analytic techniques within “research show that the RS has the best model fit when all 25-items load on one overall resilience factor instead of two factor scores” (Ahern et al., 2006; Portzky et al., 2010; Madewell and Ponce-Garcia, 2016). The internal consistency of the RS 25 (α = 0.91; Wagnild and Young, 1993; and α = 0.93; Wagnild, 2009a) has been reported to be excellent. The stability of the RS “over time (test-retest correlations ranging from 0.67 to 0.84) has been reported” (Wagnild and Young, 1993). “The RS has been validated into various languages and the internal consistency of the Russian (Aroian et al., 1997), Spanish (Heilemann et al., 2003; Sánchez-Teruel and María Auxiliadora, 2016), Swedish (Nygren et al., 2005), Japanese (Nishi et al., 2010), and Nigerian (Abiola and Udofia, 2011) versions has also been reported as acceptable (α between 0.83 and 0.93).”

## Short Version RS-14

While looking for a useful and valid instrument, not only needed for different populations but also in which the proposed factor structure can be confirmed, two major goals were in focus. The goal in focus was the need for an age-appropriate measurement of resilience suitable for adolescents and young adults. “The RS-14 demonstrates the brevity, readability, and ease of scoring that have been identified as important characteristics when selecting instruments for use with adolescents” (Pritzker and Minter, 2014, p. 332). The RS-14 “will also provide details of the pattern and profile of resilience utilizing a widely available measure of resilience which in turn will enable comparisons with previous and future research,” and therefore “will provide supporting evidence that it is a psychometrically sound measure to assess individual resilience within the age groups of adolescents and young adults” (Wagnild, 2009a; Pritzker and Minter, 2014).

Looking for more economic variation of the Resilience Scale, decreasing completion time, and designing more specifically for use with young people, Wagnild (2009a) modified the RS-25 to14 items. The brief “RS-14 scale consists of 14 self-report items measured along a 7-point rating scale ranging from ‘1—strongly disagree’ to ‘7—strongly agree.’ Higher scores are indicative of resilience level. According to the authors, scores are calculated by a summation of response values for each item, thus enabling scores to range from 14 to 98.” Scores below 65 indicate low resilience; between 65 and 81 show moderate resilience; above 81 will be interpreted as high levels of resilience (Wagnild and Young, 1993; Wagnild, 2009b, 2014).

Using principal components analyses supported a single-factor solution; remaining in the RS-14 scale were those items with all item factor loadings >0.40. Reported psychometric properties of the RS-14 have demonstrated sound psychometric properties comparable to those of the RS-25: evidence of a one-factor structure was found and high reliability (coefficient Cronbach's alpha = 0.90 and greater 0.96) and a strong correlation with the full version (*r* = 0.97, *p* = 0.001) were obtained (Wagnild, 2014). The overall factorability of the RS-14 demonstrated a robust one-factor measure of resilience, which has been replicated and has been confirmed in different studies and in the adaptations of this version for different countries (Wagnild, 2014). For instance: German α = 0.91 (Schumacher et al., 2005); Portugal α = 0.82 (Oliveira et al., 2015); Finland α = 0.87 (Losoi et al., 2013); Japan α = 0.88 (Nishi et al., 2010); China α = 0.92 (Tian and Hong, 2013); Korean α = 0.90 (Kwon and Kwon, 2014); Spain α = 0.79 (Heilemann et al., 2003); Italian α = 0.88 (Callegari et al., 2016); and Greek α = 0.89 (Ntountoulaki et al., 2017). Moreover, Yang et al. (2012) “examined the measurement invariance of the RS−14 in samples of U.S., Chinese, and Taiwanese college students and supported a one-factor model that demonstrated scalar invariance across cultures” (Yang et al., 2012). The short version RS-14 has been tested regarding its structure and it was found that results are not always totally consistent. Some discrepancies exist between findings of different studies; for instance the Brazilian version with 13 items (Damásio et al., 2011) or 12 items in the Portuguese adaptation for adolescents (Oliveira et al., 2015), and in the German Version 11 items (Schumacher et al., 2005). These discrepancies can eventually result from sampling issues: some studies used participants from very different developmental phases (Damásio et al., 2011), and others used participants <13 years old, an option that is not appropriate given that the authors of the RS advise against the use of the scale with participants from earlier ages (Wagnild, 2009b; Pritzker and Minter, 2014).

Criterion validity of the RS-14 construct in adolescent and youth groups, measured by using personal resources concepts “(such as self-esteem, self-efficacy, social support, life satisfaction and meaning in life) and those regarding indexes of psychological distress (such as depression, anxiety, stress, and individual disability)” have showed confirming findings for resilience (Nishi et al., 2010; Salazar-Pousada et al., 2010; Damásio et al., 2011; Kwon and Kwon, 2014; Pritzker and Minter, 2014; Aiena et al., 2015).

In Poland, to measure resiliency [Pol.: sprezystość psychiczna], researchers started to adapt a different resilience scale in the 1990s (Uchnast, 1998). Initially most used was the Ego Resiliency Scale (Block and Kremen, 1996). This scale was validated for adults and is known as the Psychological Resilience Questionnaire (PRQ) which is the Polish adaptation of the Ego Resiliency Scale (Kaczmarek, 2011). “It examines psychological resilience understood as a personality feature which reflects the ability to adjust the level of self-control to the demands of a situation.” At the moment, most in use is the Polish version of the Ego Resiliency Scale (Kaczmarek, 2011) and SPP-18 (Psychological Resilience Scale) for children and adolescents (Oginska-Bulik and Juczynski, 2011). In this scale, the resilience was defined in the context of four psychosocial factors. The RS-25 and the short version RS-14 are not validated for Polish language. Due to the importance of scale (reporting in many studies and confirmed scale properties) we decided to adapt it in Polish conditions.

## General Aim of the Study

The purpose of this study is two-fold. Firstly, the study attempts to assess systematically the psychometric properties of the RS-14, as proposed by Wagnild (2009a) and Wagnild (2014) The purpose of this study is two-fold. Firstly), with a large sample of Polish young people, aged 13-27, regarding three different populations in Poland: adolescents (13–17 years old), young adults (aged 19–27), and groups of adolescents having specific psychosocial needs or possessing conditions of maladjustment (aged 13–18). Secondly, the study explores the validity of the RS-14 as a measurement of resilience in adolescents who have special needs or conditions of maladjustment who are in institutions: those who are in residential socio-therapeutic and educational treatment centers, and those who came into contact with the Polish justice system and remain in correctional custody in a juvenile facility. Although there have been several validations of the RS-14 Scale for adolescents conducted worldwide, nowhere has it been previously explored with young people having special needs or possessing conditions of maladjustment.

The research interest for specificity of this group is primarily related not so much to institutional determinants but to the psychosocial and behavioral characteristics of the young people staying there. They were remanded to various forms of educational, therapeutic resocialization centers as a result of professional diagnosis and legal rulings because of their impairments or abnormalities. Such placements and interruptions of the regular life environment of adolescents, despite their special needs or maladjustment difficulties, can have an additional impact on their psychosocial functioning and the quality of custodial care. “This group of adolescents can be considered a vulnerable population” (Seita and Brown, 2010; Oginska-Bulik and Kobylarczyk, 2016). Those adolescents may “experience learning disabilities, have also a higher risk of school failure, and mental and behavioral problems in much greater proportions than their peers” (Quinn et al., 2005; Bruce et al., 2010). There is also danger of the “development of or the reinforcement of depression, anger, lack of social-emotional skills, and mental anguish.” Often, “they face significant challenges and difficulties throughout their lives, with their families, schools, friends, and peers that finally may lead to the development of behavioral problems and can impede their developmental well-being” (Avanci et al., 2007; Mastropieri and Scruggs, 2010; Scruggs et al., 2010; Pecora, 2012). The successful identification of personal resilient aspects of “young people with those particular needs, vulnerabilities, and impairments within the custodial and correctional environment may mean that it is possible to target additional support and intervention efforts to those in most need.” Although the institutional context of the custody is beyond the scope of the current study, it brings the possibility to explain whether the RS-14 is a valid tool for assessment of personal and individual aspects of resilience related to that specific population of adolescents with special needs, with problematic, externalizing educational, cognitive-behavioral and socioemotional impairments, and also related to the population of adolescents who are juvenile offenders.

## Design of Studies

This study was designed and performed as four partial studies analyzing different aspects of validation for the Polish version of RS-14 questionnaire in tested populations. Study 1 was dedicated to the procedure of developing the Polish language version. Study 2 consisted of validity and reliability testing of the Polish RS-14 with three samples. Using exploratory and confirmatory factor-analyses, the factor structure and construct validity were evaluated. Study 3 applied the test-retest measurement based on one population of adults in order to verify stability in a 4-weeks reliability of the RS-14 Scale. Study 4 assessed validity by investigating factors linked to resilience and mental health outcomes. Scores on positive psychological variables (satisfaction with life) and indexes of psychological distress (depression and perceived stress) were used to verify current validity of RS-14.

## Study 1. Development of the Polish Language Version of Resilience Scale 14

Development of the Polish language version of the RS 14 was prepared by following the procedures from the Cross-Cultural Survey Guidelines (Harkness et al., 2010) and World Health Organization WHO (Erkut, 2010) and having support, proceeded in several stages, from author of the tool Wagnild. The scale was translated into Polish by four independent translators under the supervision of an expert in research methodology and psychology. The scale was translated into Polish with great attention to accuracy, although—for the semantic accuracy of the content—some modifications were made where the faithful translation was not possible. Finally, measures were taken to prepare a version of the translation that captures the essence of the content of the tested construct and stylistically better fulfills the requirements of the Polish language. The Polish translation, agreed upon by a team of experts, was subjected to a reverse translation from Polish back into English, performed by two independent native English speakers. The new English version was compared with the original Wagnild (2009a,b) English version, and the translators assessed that there were no significant differences between the original and back-translated English versions. Some problems occurred in the case of statements containing idiomatic phrases, but that is understandable due to the nature of translation. This kind of operation was consulted with the author of the tool.

Efforts were also made to provide a possibly good fit of the Polish language version to the age group of persons who are representatives of the population for which the tool was translated. For this reason, performed was an assessment of preliminary understanding of the Polish scale version in a group of 10 people aged 18–30. First Respondent R1—18 year old man; R2—19 year old man, secondary education; R2—25 year old woman, secondary education; R3—30 year old man, primary education; R4—27 year old man, higher education; R5—24 year old man, higher education; R6—25 year old woman, higher education; R7—29 year old man, secondary education; R8—27 year old woman, higher education; R9—30 year old woman, higher education; R10—24 year old woman, secondary education. “When considering the face validity of the measure for use with adolescents with special needs and offenders, it was also important to determine if the language used in many of the items would appear simple and appropriate for that population. Twenty adolescents were asked to take part in preliminary language work. However, some items may benefit from further simplification to help maximize comprehensibility (e.g., the item ‘I have self-discipline’)” (Windle et al., 2011).

The next step involved the interviewing of a group and the handing in of the translated version of the tool. The task of the respondents was to tick “yes” when the sentence was fully understood, and “no” if it was incomprehensible or raised doubts. Each question was understood by the respondents. There were no problems with understanding given statements. After that, competent judges and a team of experts compared the original version with both versions of retranslation and made necessary amendments in the Polish translation in order to reflect fully the authors' intentions and the basic content of each item of the method. After the above-mentioned procedures, the content validity was recognized by the method of examining the compliance of expert opinions. To this end, three experts were appointed to analyze the clarity and comprehensibility of the items for pressure measurement according to the construct definition. In this respect, experts were asked to determine whether the listed items refer to the contents of the tested construct. Calculated content validity ratio was 0.93 (estimated by the W Kendalla test), which indicates high clarity and comprehensibility of the analyzed construct.

## Study 2: Exploratory and Confirmatory Factor Analysis

### Objective

In response to fulfill the need for a brief measure of resilience, a Polish version of the RS-14, which would be psychometrically validated and across diverse populations, it was important to examine the measure's central tendencies, internal consistency and factor structure across the three chosen subgroups including the gender and age of adolescents, young adults, and those with special needs. “We hypothesized that for all three samples, a one-factor model would be supported and that scores would be highly reliable as assessed by conventional interpretive standards” (Aiena et al., 2015).

### Participants and Procedure

Analyses were based on three main samples, one made up of adolescents in age of 13–17, the second involved young adults in age 19–27, and the third included individuals in age of 13-18 with adaptive psychosocial difficulties who were in custody of educational, therapeutic or correctional institutions. The total sample included 2,715 young people from three different groups within Poland, aged from 13 to 27 (*M* = 19.79, *SD* = 3.90), predominately female (69.09%).

Sample 1—adolescents (*N* = 400). The sample included adolescents coming from 20 different junior high-schools in the voivodeship Podlaskie. The sample was with age ranging from 13 to 17 years old (*M* = 14.22, *SD* = 0.86) predominately female (58.3%). Sample 2—early adulthood (*N* = 1659). Data were also gathered at a medium-sized university located in northeast Poland. Overall, we had results from students aged 19–27 years old (*M* = 22.56, *SD* = 1.82) predominately female (82.3%). Sample 3—problem group (*N* = 656). The sample consisted of three groups: socially maladjusted juveniles from probation centers was (*N* = 116) with age ranging from 13 to 17 years old (*M* = 15.01, *SD* = 1.48) predominately male (75.0%), adolescents with psycho-social impairments attending (staying) in Youth Sociotherapy Centers (*N* = 293, aged 13–18 years old, *M* = 16.02, *SD* = 1.22) and adolescents externalizing educational difficulties (Youth Educational Centers, *N* = 247, with age ranging from 13 to 18 years old (*M* = 16.53, *SD* = 0.98). The selection of the third study sample was purposive due to the specifics of the facilities to which the studied individuals were admitted. The main criterion for including respondents to sample 3 was the occurrence of emotional and behavioral disorders. A comparative analysis using ANOVA did not confirm differences between problem groups (youth from probation centers, educational centers and sociotherapy centers) in terms of the RS-14 index [*F*_(2, 653)_ = 2.63; *p* > 0.05].

Sociotherapeutic and educational centers are facilities outside the place of residence, which function around the clock. In Poland there are currently over 3,000 youth aged 13–18 who are staying in such Youth Sociotherapy Centers and over 4,000 who are staying in Youth Educational Centers. Youth Sociotherapy Centers are designed for adolescents who, due to developmental disorders, learning difficulties, and disorders in social functioning, are at risk of social maladjustment and require a special organization of learning, methods of work, education and sociotherapy. Currently, there are 74 in Poland. Youth Educational Centers are designed for socially maladjusted youth who require the use of a special organization of learning, methods of work and education. Currently, there are 96 in Poland. A Probation Center is a day-care facility that provides prevention, care, education, rehabilitation, and therapeutic activities. The activities undertaken at the center are aimed at changing juvenile attitudes toward the socially desirable, ensuring the optimal development of their personality. Probation centers operate under the district courts. Currently, there are 97 in Poland. At the present time, ~1,000 juveniles have been referred to such facilities.

### Data-Analytic Strategy

Psychometric properties of the RS-14 were assessed using univariate and bivariate analyses conducted with the statistical software IBM SPSS Statistics and IBM AMOS. The Cronbach Alpha coefficient was used to characterize coherence (dimension of reliability). In the case of time stability assessment, Pearson's r correlation analysis and the t test for dependent samples were used. Principal component analysis was applied to verify the single factor structure of RS-14 reported by Wagnild and Young (1993). Exploratory and confirmatory factor-analytic procedures were employed to assess the factor structure of the measure. The factor accuracy was analyzed using EFA (main component method, oblimin rotation) and CFA (IBM AMOS, ML method). The models from the EFA were tested for an adequate fit using the following fit indexes: chi-square and degrees of freedom, RMSEA including 90% confidence interval (CI); (Steiger, 1990) SRMR, the Tucker–Lewis Index (TLI); (Tucker and Lewis, 1973), CFI; and GFI (Hu and Bentler, 1999).

### Item Analysis

Table 1 shows the basic descriptive statistics for the analyzed items and the IDI (Item Difficulty Index) value. It was not observed that the distribution of analyzed items differed significantly from the normal distribution. In addition, IDI index did not confirm the existence of floor or ceiling effects in the data (only in the case of item 9 “I keep interested in things” did a slight ceiling effect show for all three samples). Frequency analysis for individual test items showed no problems with data granulation. Due to that, all items were included in further analyses. This may be due to response set bias, particularly social desirability and acquiescence.

**Table 1:** Descriptive statistics and IDI value for individual test items.

	**Early adulthood (*****N*** **=** **1,659)**					**Adolescents (*****N*** **=** **400)**					**Problem group (*****N*** **=** **656)**				
**RS14**	***M***	***SD***	**Skewness**	**Kurtosis**	**IDI**	***M***	***SD***	**Skewness**	**Kurtosis**	**IDI**	***M***	***SD***	**Skewness**	**Kurtosis**	**IDI**
Item 1	5.50	1.13	−0.84	1.17	0.78	5.00	1.32	−0.66	0.40	0.71	5.04	1.53	−0.46	−0.40	0.72
Item 2	4.97	1.51	−0.54	−0.23	0.70	5.02	1.58	−0.52	−0.39	0.72	5.22	1.75	−0.80	−0.23	0.75
Item 3	4.26	1.56	−0.17	−0.63	0.60	4.46	1.60	−0.39	−0.45	0.64	4.34	1.87	−0.27	−0.92	0.62
Item 4	4.82	1.61	−0.54	−0.38	0.68	5.16	1.81	−0.75	−0.38	0.74	5.15	1.91	−0.79	−0.46	0.74
Item 5	5.12	1.35	−0.68	0.18	0.73	4.53	1.56	−0.36	−0.35	0.65	4.63	1.72	−0.50	−0.43	0.66
Item 6	5.06	1.37	−0.57	0.07	0.72	4.65	1.57	−0.41	−0.25	0.66	4.56	1.74	−0.41	−0.51	0.65
Item 7	5.02	1.41	−0.45	−0.16	0.71	4.71	1.60	−0.38	−0.40	0.67	5.30	1.69	−0.90	0.08	0.76
Item 8	4.81	1.57	−0.44	−0.51	0.68	4.59	1.53	−0.39	−0.26	0.66	4.61	1.78	−0.49	−0.54	0.66
Item 9	5.60	1.42	−1.03	0.64	0.80	5.83	1.50	−1.29	0.94	0.83	5.84	1.57	−1.41	1.32	0.83
Item 10	5.55	1.36	−0.88	0.30	0.79	5.67	1.47	−1.12	0.87	0.81	5.61	1.60	−1.12	0.55	0.80
Item 11	4.59	1.62	−0.35	−0.59	0.65	4.94	1.62	−0.63	−0.20	0.71	5.12	1.76	−0.72	−0.34	0.73
Item 12	5.93	1.04	−1.18	2.18	0.84	5.51	1.45	−1.09	0.94	0.79	5.69	1.48	−1.19	0.98	0.81
Item 13	5.52	1.59	−1.03	0.34	0.78	5.39	1.78	−0.91	−0.16	0.77	5.26	1.87	−0.83	−0.39	0.75
Item 14	5.22	1.12	−0.51	0.47	0.74	5.03	1.39	−0.71	0.50	0.72	5.06	1.65	−0.71	−0.19	0.72

### Factorial Validity

By applying the Velicer MAP method, it was found that the optimal number of components to be extracted is one, which confirms the original factor structure of the questionnaire. The effect was observed both when the results of the interviewees were analyzed together and when they were divided into validation groups. Subsequently, the result obtained by MAP was verified by means of principal component analysis with oblimin rotation. As the criterion for the number of factors to be isolated, the eigenvalue was set to be equal to at least 2. Assumptions of factor analysis were met [KMO = 0.908; ${\text{chi}}_{\left(91\right)}^{2}$ = 10028.76; *p* < 0.001]. The created factor explains a total of 35.02% variance of the questionnaire. The factor structure was checked for the three validation groups, there were no significant differences in the size of explained variance: early adulthood (38.51%), young adolescence (34.62%), problem group (31.11%). The obtained factorial results correspond to the expected one factor dimensions of the original version of the Questionnaire.

Parallel analysis indicated that a one-factor solution was the most appropriate for all three the samples. As indicated in Table 2, the one-factor model showed good item loadings and similar results were observed regarding the item loadings across the three samples. All items loaded saliently on this factor, with item loading ranged between 0.505 and 0.719 in the early adulthood sample. In the adolescents it was between 0.464 and 0.770 and in the problem group it was between 0.400 and 0.668. The lowest loadings were still significant, confirming the coherent structure of the Polish version of the instrument.

**Table 2:** Fourteen-item Resilience Scale (RS−14) factor loadings.

	**Early adulthood** ** (*N* = 1,659)**	**Adolescents** ** (*N* = 400)**	**Problem group** ** (*N* = 656)**
Item 1	0.544	0.602	0.638
Item 2	0.657	0.681	0.525
Item 3	0.505	0.464	0.400
Item 4	0.561	0.711	0.563
Item 5	0.576	0.664	0.521
Item 6	0.514	0.646	0.397
Item 7	0.521	0.497	0.582
Item 8	0.542	0.555	0.541
Item 9	0.539	0.495	0.518
Item 10	0.561	0.595	0.578
Item 11	0.673	0.770	0.668
Item 12	0.620	0.485	0.527
Item 13	0.655	0.682	0.628
Item 14	0.719	0.734	0.645

The next step included comparing the results of the model fitting measures from other available studies. There were no significant differences between the model fitting scores obtained in the confirmatory factor analysis (CFA). The Polish version of RS-14 showed a coherent one-dimensional factor structure with remarkable stability across the three samples.

Additionally, it should be noted that in the three sample validation groups no major differences were observed. Detailed results are provided in Table 3.

**Table 3:** Comparison of model fit measures (CFA) for univariate solution for RS14 questionnaire.

	**Estimation**	**Model fit measures**							
		**chi2**	**Df**	**RMSEA**	**90% CI**	**SRMR**	**TLI**	**CFI**	**GFI**
Current study—total sample (*N* = 2,715)	ML	31.37	13	0.023	[0.013;0.033]	0.019	0.98	0.99	0.99
Current study—early adulthood (*N* = 1659)	ML	30.62	22	0.015	[0.001;0.027]	0.024	0.99	0.99	0.99
Current study—adolescents (*N* = 400)	ML	43.06	22	0.049	[0.027;0.071]	0.060	0.94	0.98	0.98
Current study—problem group (*N* = 656)	ML	34.63	22	0.042	[0.001;0.101]	0.098	0.93	0.98	0.97
(Aiena et al., 2015) (students, *N* = 882)	ML	893.47	77	0.11	[0.10;0.12]	0.04	0.92	0.93	x
(Losoi et al., 2013) (students, *N* = 243)	x	X	X	0.101	X	X	x	0.94	0.86
(Damásio et al., 2011) (students and adults, *N* = 1,139)	ML	19219	65	0.59	[0.049;0.065]	0.041	0.91	0.93	x

*X, no information in the article*.

### Cross-Group Validity Reliability of Scales

Cronbach's alpha coefficient was used to examine the internal consistency of the obtained questionnaire indicators. The results are presented below.

The reliability of the created factor was 0.853 (total sample) in the validation trial, which confirms its high consistency. After division into validation groups, no significant differences in scale consistency were observed. Cronbach's alphas ranged between 0.824 and 0.871 across the three samples:

- Early adulthood: α = 0.871 [ICC = 0.871; *F*_(1658,211554)_ = 7.74; *p* < 0.001],
- Adolescents: α = 0.851 [ICC = 0,851; *F*_(399,5187)_ = 6.71; *p* < 0.001],
- Problem group: α = 0.824 [ICC = 0.824; *F*_(655,8515)_ = 5.68; *p* < 0.001],

The highest reliability of the obtained factor was by the young adults (α = 0.871) and respectively lowest by the problem group: (α = 0.824).

Therefore, the internal consistency reliability of the RS-14 is not only acceptable across all sample populations, but also rather robust. Additionally, the analysis of the discriminative power of individual items shows that all test items are positively correlated with the scale. Because of the slight variation in Cronbach's Alpha value for items where the correlation with the scale was <0.300, it was decided to include all test items in the factor. The exact results are shown in Table 4 below.

**Table 4:** Discriminatory power values for items included in the factor.

	**Early adulthood (*****N*** **=** **1,659)**		**Adolescents (*****N*** **=** **400)**		**Problem group (*****N*** **=** **656)**	
	**Item correlation total**	**Cronbach's alpha**	**Item correlation total**	**Cronbach's alpha**	**Item correlation total**	**Cronbach's alpha**
Item 1	0.518	0.864	0.451	0.844	0.533	0.808
Item 2	0.605	0.859	0.574	0.836	0.433	0.814
Item 3	0.389	0.871	0.420	0.846	0.323	0.823
Item 4	0.635	0.857	0.467	0.844	0.461	0.812
Item 5	0.582	0.860	0.488	0.842	0.433	0.814
Item 6	0.564	0.861	0.425	0.845	0.315	0.822
Item 7	0.415	0.869	0.432	0.845	0.474	0.811
Item 8	0.475	0.866	0.459	0.843	0.449	0.813
Item 9	0.423	0.868	0.447	0.844	0.415	0.815
Item 10	0.515	0.863	0.471	0.843	0.472	0.812
Item 11	0.699	0.853	0.583	0.836	0.564	0.805
Item 12	0.408	0.868	0.530	0.839	0.424	0.815
Item 13	0.597	0.859	0.566	0.837	0.518	0.808
Item 14	0.658	0.858	0.633	0.834	0.541	0.807

### Descriptive Statistics of the Indicators

Table 5 presents the basic descriptive statistics for created indicator based on the validation samples. In addition, two distribution measures (skewness and kurtosis) showed that the distribution of the two formed indicators does not differ from the normal distribution. There is no reason to infer that there are differences between the analyzed validation samples in terms of the mean of RS-14 indicator.

**Table 5:** Descriptive statistics of the RS14 indicator.

	**Early adulthood** ** (*N* = 1,659)**	**Adolescents** ** (*N* = 400)**	**Problem group** ** (*N* = 656)**
M	71.97	70.48	71.43
Me	73.00	71.00	73.00
SD	12.13	12.75	13.22
Skewness	−0.62	−0.54	−0.51
Kurtosis	0.64	0.56	0.31
Min	14	17	17
Max	98	97	98

Responses to the RS-14 tended to be negatively skewed. Most respondents scored in the upper range of possible scores - range from 14 to 98 (*M* = 73.00; *SD* = 12.3) for the sample of early adulthood.

The total mean score on the RS-14 was respectively 71.97 adulthood, 70.48 adolescents and 71.43 problem group Resilience scores for men (*M* = 71.51; *SD* = 12.85) and women (*M* = 71.67; *SD* = 12.35) did not differ significantly, with Mann-Whitney *U-*test = 7,67,093 and *p* = 0.599. Similarly, no difference between resilience scores was found between the mean age groups of 13–17 and 18–27 years (*U* = 833081.5; *p* = 0.062). The ANOVA did not confirm the existence of differences between the analyzed validation groups [*F*_(2, 2712)_ = 2.38; *p* = 0.093; eta^2^ = 0.042]. Low value of the eta-square index indicates a small effect of dependence between variables.

### The Relationship Between Indicators and Metrics—Comparison Between Students (Early Adulthood Group), Adolescents, and Problem Group

By means of analysis with Mann Whitney's *U*-test and the t test for independent samples, it was investigated whether there was a dependency between the constructed indicator and the sex of the respondents. It was not confirmed that RS indicator value differs between and men in any of the analyzed validation groups:

- Early adulthood: *Z* = 0.57; *p* > 0.05,
- Adolescents: *t*_(398)_ = 0.09; *p* > 0.05,
- Problem group: *t*_(654)_ = 1.05; *p* > 0.05,

In addition, the Pearson's r correlation analysis confirmed the existence of relations only in the group of early adulthood—with the age of the subjects observed was growth of the RS indicator (*r* = 0.120, *p* < 0.001). For the remaining validation groups, correlation with age was statistically insignificant.

## Study 3. Test-Retest Reliability of the RS-14

The aim of study 3 was to evaluate the test-retest reliability of the Polish version of the RS-14.

### Participants and Procedure

Assessment of stability was carried out in a group of 42 participants (pedagogy students) (59.1% females; ages ranged from 20 to 24 years of age, *M* = 22.02, *SD* = 1.04) from Univeristy of Bialystok. The second test was performed 4 weeks after the first one. For the tested subgroup of 42 participants who had participated in the second round of testing, 2 measures were applied: t-student and the Intraclass Correlation Coefficient (ICC) were calculated to evaluate the test-retest reliability. The commonly cited cutoff for ICC considers good values between 0.60 and 0.74 (Cicchetti, 1994).

## Results

In order to check the stability of the adapted tool, an analysis was conducted in the group, using t-Student test for dependent samples. The analysis showed that at the second measurement the participants did not differ significantly, which shows that the scale is stable; *t*_(41)_ = 1.57; *p* > 0.50. Obtained coefficient of absolute stability (correlation between test and retest) shows high level of time stability of the RS-14 result; *r*_(40)_ = 0.88; *p* < 0.001. The research indicates a high time stability of the overall RS-14 result. We can therefore conclude that the Scale is an available construct relatively independent of situational influences. High stability was also confirmed by the ICC indicator (Intraclass correlation coefficient)—using the absolute compliance method, a high ICC was observed [ICC = 0.933; *F*_(41, 41)_ = 15.45; *p* < 0.001].

## Study 4. Criterion Validity

The aim of study 4 was to evaluate validity of the Polsih version of the RS-14.

### Participants and Procedure

It was important to focus on two groups: one representing young adults, considered relatively “low-risk” for psychological distress and the second sample a problem group with respectively higher contextual stressors. Sample 1. A sample (*n* = 382) of undergraduate and post-graduate students from the University of Bialystok and Cardinal Stefan Wyszynski University in Warsaw was recruited. They were between the ages of 19 and 27 years of age (*M* = 25.60, *SD* = 7.45). The individual questionnaires in the questionnaire package were arranged in the following order: (1) RS-14, (2) SWLS, (3) KADS, (4) PSS. The sample consisted of 84.1% females. Sample 2. The sample of juveniles from Youth Educational Centers was (*N* = 120) with age ranging from 13 to 18 years old (*M* = 16.22, *SD* = 1.07) and predominately male (56.9%). This study was conducted on a different samples as noted earlier. The individual questionnaires in the questionnaire package were arranged in the following order: (1) RS-14, (2) SWLS, (3) KADS.

## Measures

### Satisfaction With Life

Life satisfaction (Diener et al., 1985) “was measured with The Satisfaction with Life Scale (SWLS), developed by Diener et al. (1985) and adapted by Juczynski (1999), which assesses the cognitive aspect of SWB. The SWLS consists of five items rated by a respondent using a seven-point scale, ranging from ‘strongly disagree’ (1) to ‘strongly agree’ (7). Items are summed to give a total score ranging from 5 (low satisfaction) to 35 (high satisfaction). Sample items include “I am satisfied with the conditions of my life” and “So far, I have gotten the important things I want in life.” The Polish version of the SWLS had shown test-retest reliability (0.86), internal consistency—Cronbach's alpha (0.81), and discriminant validity (up 0.50)” (Juczynski, 1999).

### Depression

The Kutcher Adolescent Depression Scale (KADS) (Brooks et al., 2003) “is a commonly used screening test used to identify young people at risk for depression. It is a self-report scale specifically designed to diagnosis and assess the severity of adolescent depression. The KADS consists of six items rated by a respondent using a four-point scale, ranging from ‘hardly ever’ (0) to ‘all of the time’ (3). Validation of the Polish version of KADS in a group of students aged 18–24 years has shown its high reliability and content validity. Confirmatory factor analysis showed the good fit of model to empirical data: SB $\chi_{\left(15\right)}^{2}$ = 968.688, *p* < 0.001, RMSEA = 0.053, CFI = 0.958, SRMR = 0.029. Factor loading ranged from 0.40 to 0.80. Total score ranged from 0 to 18” (Mojs et al., 2015).

### Stress

The Perceived Stress Scale (PSS) “is the most widely used psychological instrument for measuring the perception of stress (Cohen et al., 1983). This scale is designed to assess the degree to which respondents find their lives unpredictable, uncontrollable, and overloading. It uses a 10-item measure of the degree to which situations in one's life are appraised as stressful. Total scale score is calculated by reverse-scoring positively worded items and then summing all 10 items. The potential range of values for the total scale score is 0–40. Reported reliability is 0.91 in college and community samples and 0.88 in a sample with early adolescents (Yarcheski and Mahon, 1999). The PSS has been used in a range of settings and has been shown to relate to a number of psychological responses, including anxiety and depressive symptoms. The Polish version of the PSS had shown test-retest reliability (0.72), internal consistency—Cronbach's alpha (0.86)” (Juczynski and Oginska-Bulik, 2012).

### Construct Validity

To assess for validity support, correlations were calculated between the RS−14 and the satisfaction-with-life scale SWLS, as well as with the three subscales of the depression scale KADS and with the Perceived Stress Scale (PSS) in the sample of young adults and in the sample of the group with special needs. Results are shown in Table 6.

**Table 6:** The correlation coefficients of linear Pearson's r RS-14 and SWLS, KADS, PSS.

	**KADS**	**Sadness**	**Lack of** ** faith**	**Physical** ** exhaustion**	**Sense of** ** hardness** ** of life**	**Worries**	**Suicide of** ** thoughts**	**PSS**	**SWLS**
RS14 young adults	−0.58^**^	−0.51^**^	−0.54^**^	−0.45^**^	−0.52^**^	−0.46^**^	−0.27^**^	−0.56^**^	0.66^**^
RS14 problem group	−0.34^**^	−0.13	−0.23^*^	−0.26^**^	−0.27^**^	−0.24^*^	−0.36^**^	x	0.63

** *p < 0.01*.

Firstly, as expected, life satisfaction was positively and significantly associated with resilience (RS-14) in both the young adults and special needs groups. Secondly, depression and perceived stress (PSS) were negatively and significantly correlated with resilience in the young adults group. Moreover, negative correlations were observed regarding the dimension-of-depression scale KADS (sadness, lack of faith, physical exhaustion, sense of hardness of life, worries, and suicide of thoughts). Thirdly, depression was negatively and moderately correlated with resilience in the group with special needs.

## Discussion

Given the impact of resilience in the personal and social development of youth, and given the relevant increasing interest in resilience findings in theory and practice, this current study was impelled to report relevant validation statistics using the measures of the Scale RS-14 (Wagnild, 2009a) within the Polish population of young people. This methodological research examined the concept of resilience by validating the RS-14 with an average population of adolescents (aged 13–17 years) and young adults (age 19–27 years) and secondly by exploring validation in the case of young people with special needs and socially maladjusted offenders who are in educational, therapeutic, probation centers (aged 13–18 years). This has been lacking to date, and therefore this lack brings support for diagnostics in this field both in Poland and also in future international research regarding the RS-14 (see Table 7).

**Table 7:** The English version of RS-14 alongside the polish version of RS-14.

Item 1 Ability to cope
I usually manage one way or another
W sytuacji trudnej zazwyczaj radzȩ sobie w taki czy inny sposób
Item 2 Pride
I feel proud that I have accomplished things in life
Jestem dumny/a z moich dotychczasowych osiagniȩć
Item 3 Acceptance
I usually take things in stride
Zazwyczaj podchodze do spraw zȩ spokojem
Item 4 Self-regard
I am friends with myself
Lubie siebie
Item 5 Organized
I feel that I can handle many things at a time
Potrafie radzić sobie z wieloma rzeczami na raz
Item 6 Drive
I am determined
Jestem osoba̧ zdeterminowana̧
Item 7 Perseverance
I can get through difficult times because I've experienced difficulty before
Radzȩ sobie w trudnych chwilach, poniewaz doświadczyłem/am ich wcześniej
Item 8 Willpower
I have self-discipline
Jestem samo-zdyscyplinowany/a
Item 9 Interest/engagement
I keep interested in things
Mam swoje zainteresowania
Item 10 Humor
I can usually find something to laugh about
Zazwyczaj znajdujȩ jakiś powód do śmiechu
Item 11 Self-efficacy
My belief in myself gets me through hard times
Moja wiara w siebie pomaga mi przetrwać ciȩzkie chwile
Item 12 Dependable
In an emergency, I'm someone people can generally rely on
W nagłych sytuacjach ludzie z reguły moga̧ na mnie polegać
Item 13 Meaning
My life has meaning
Moje zycie ma znaczenie
Item 14 Resourcefulness
When I'm in a difficult situation, I can usually find my way out of it
Kiedy jestem w trudnej sytuacji, zazwyczaj znajdujȩ z niej wyjście

Confirmatory factor analysis and exploratory factor analysis indicated that the Polish version of RS-14 was characterized by good construct validity to the single factor model postulated by the authors of the scale (Wagnild, 2009b). The obtained one factorial structure and variance results correspond to the expected dimensions of the original version of the Resilience Scale 14 (a principal components factor analysis with direct oblimin rotation indicated one strong factor solution accounting for 35% of total variance) (Wagnild and Young, 1993; Wagnild, 2009b). Those results are consistent with previous studies of the RS-14 that also exhibit a consistent tendency to negative skew (Wagnild, 2009b; Pritzker and Minter, 2014). Nevertheless, in the current study “EFA's and CFA's indicated that all items loaded cleanly onto a single factor consistent with cohesive structure for a one-factor model. This model supports a resilience factor, which confirms the original factor structure proposed by Wagnild (2009a).” All three “samples showed invariance across models for sex and age, indicating that males and females in different ages report similarly on RS−14 items.” Comparable results were obtained in studies of young people in Japan (Nishi et al., 2010), where a single factor solution explained 39.4% of variance; in the Brazilian study (Damásio et al., 2011) a single factor solution explained 31.93%. Principal component analysis identifies a single factor solution accounting for 45.4% of the variance in Pritzker and Minter (2014) research. In Aiena et al. (2015) studies indicate cohesive structure for a one-factor model explaining 67.6% of the variance in the undergraduate sample. Factor analysis of 14 items revealed two factors with a total variance of 55.43% in the Kwon and Kwon (2014) study. The Spanish version (Sánchez-Teruel and María Auxiliadora, 2016) data show that the scale has a different factor structure to the original version—two factors with a total variance 75.97%. Research did not always confirm an overall good model fit while validating RS-14 when compared with other measurements of resilience. Madewell and Ponce-Garcia (2016) showed that the RS-14 achieved an adequate, but not a good model fit applying to youth during the transition age to adulthood.

As former research in other languages suggested that there was greater support for the one-factor model, as originally proposed by author of the Scale RS-14 (Wagnild, 2009a,b) and further replicated and confirmed in relevant international research (Nishi et al., 2010; Damásio et al., 2011; Losoi et al., 2013; Kwon and Kwon, 2014; Pritzker and Minter, 2014; Aiena et al., 2015), similarly, this current study confirmed the internal structure of the Scale within three Polish samples. As hypothesized, the one factor structure was found with all 14 items having factor loadings higher than (α = 0.85) in all three samples. Obtained results show that the RS-14 scale can be used in both the research and individual diagnosis within each of the analyzed validation groups. Comparable results were obtained in in studies of young people in Japan (Nishi et al., 2010). Data show that the scale has adequate internal consistency (α = 0.88). In the Brazilian study (Damásio et al., 2011) internal consistency was similar and equal α = 0.82. The Korean study (Kwon and Kwon, 2014) reached excellent α = 0.90, and similarly in the USA study (Aiena et al., 2015) excellent internal consistency (α = 0.96) was identified for adolescents aged 15–19 (α = 0.91). The Spanish version (Sánchez-Teruel and María Auxiliadora, 2016) data show that the scale has adequate internal consistency (α = 0.79).

In our study both the mean and median scores for the full sample fall within the range of “moderate resilience” (Wagnild, 2009b). Consistent with previous studies using the RS, the levels of resilience increased with age and presented no relation with gender (Lundman et al., 2007; Wagnild, 2009b; Nishi et al., 2010; Portzky et al., 2010; Losoi et al., 2013) “In terms of Wagnild's (2009a,b) scoring guidelines, the problem sample was higher overall, whereas the early adulthood sample scored in the moderate range. With respect to differences in scores by sex or age, scores did not vary more than a few points from one another within each sample.”

Those results are confirming findings of other studies proving test-retest validation. The research indicates a high time stability of the overall RS-14 result (0.88). Though there are not many of them, the Test-retest for young persons (correlation between test and retest) was in the Japanese research Nishi et al. (2010) 0.84 and in the Chinese study Tian and Hong (2013) 0.70 and in the Italian study 0.60–0.70 (Callegari et al., 2016). Although this current study demonstrates a very good test-retest reliability of the Polish RS-14 version, further research about stability is needed. The number of participants was relatively small; therefore, measurement should be made of cross-group differences, e.g., the group with special needs was not measured.

The criterion validity used in this current study has shown and confirmed the RS-14 theoretical construct to be a very good, reliable and valid construct. Validity concept being measured as positive correlations between RS-14 scores and satisfaction with life (SWLS) (Grotberg, 1995; Cohn et al., 2009). RS-14 scores was inversely associated with stress (PSS) and depression (KADS). These findings have also been reported by the original scale RS-14 (Wagnild, 2009a) and studies regarding validation of RS-14 in other countries (Castillo and Dias, 2009; Damásio et al., 2011, p. 14; Kwon and Kwon, 2014; Aiena et al., 2015; Callegari et al., 2016; Ntountoulaki et al., 2017).

“These findings are consistent with a range of studies that note resilience's significant positive relationships with other adaptive concepts, as well as a significant negative relationship with psychological distress” (Fredrickson et al., 2003; Tugade and Fredrickson, 2004; Nishi et al., 2010; Damásio et al., 2011; Scali et al., 2012; Aiena et al., 2015). Obtained results investigating validity are consistent with these findings “with a range of studies that note resilience's significant positive relationships with other adaptive concepts, as well as a significant negative relationship with psychological distress.” “Resilient persons are thought to have the ability to be less vulnerable to depression and to perceive their life satisfaction with less stress. Thus, these results are consistent with the concept of resilience.” Those results replicate measurement reported originally by Wagnild (2009b) which found RS-14 to be positively correlated with life satisfaction, and inversely related to depression. The same results were obtained by validation studies of the original English and from other countries' versions of RS-14 done with regular populations (in Portuguese: Castillo and Dias, 2009; Italian: Callegari et al., 2016; American: Aiena et al., 2015; Korean: Kwon and Kwon, 2014; Japan: Nishi et al., 2010).

This current study found that the RS-14 offers promise as a psychometrically strong and cross-group valid brief measure of resilience for use not only with both early and middle adolescents and young adults, but also with those of special needs who may experience adverse conditions, especially when they are qualified in special needs groups. Through multi-group analysis, this current study showed that the one-factor model was similar between males and females, younger and older adolescents, and young adults; and the one-factor model to be broadly similar among those with special needs or externalizing offending behavior. From a theoretical vantage point the study results are of a new assessment of resilience within Polish young people, regardless of gender, age, and specificity of individual and institutional determinants. At the same time, they are also promising for use to assess those being in educational care. This is obviously an exploratory approach and should be continued in the course of further research.

Setting aside the discussion of whether resilience will be understood as a relatively constant, stable feature of personality and trait-like characteristics or as a dynamic process and skills-oriented approach (Montpetit et al., 2010), all nevertheless could be “associated with psychological resilience inclusive to the use of active and adaptive coping strategies” (Southwick et al., 2005). “At the same time, we must consider that not everyone who uses coping skills is resilient. Some attempts to cope are not successful, and if the coping skill does not lead to a good outcome, the person is not resilient” (Beasley et al., 2003; Campbell-Sills et al., 2006; Fougere and Daffern, 2011; Fletcher and Sarkar, 2013). “According to Rutter (2012) resilience stresses the positive aspects and the potential to overcome, that is, the capacity the individual has of constructing new paths, of recovering his/her development from the breaking point on and of reconstructing him/herself. As it is, being resilient implies the development of competences, despite the experience of adverse situations” (Rutter, 1999, 2012). That is why the individual's capacities of resilience, in the opinion of other scholars is considered more “a personality characteristic that moderates the negative effects of stress and promotes adaptation.” That is understood as the central role for the ability to cope successfully with change or misfortune.

A person “being classed as ‘resilient’ based on one or more of these adaptive outcome measures does not imply resilience across all domains or contexts.” Specifically, one person may display resilience at one domain of competence, for instance at academic achievement, but does not at another domain, such as in health or social risk behaviors (Jaffee and Gallop, 2007). For example, in other studies “fostered youth scored similar levels of depression and self-esteem compared to non-fostered youth, but revealed discrepancies in other adaptive domains (i.e., educational)” (Wright and Masten, 2005; Luthar, 2006; Surtees et al., 2006; Jain and Cohen, 2013; Zuill, 2016). Therefore, it is important to differentiate “the resilience subtype and context under investigation, e.g., psychological resilience, educational resilience, foster and custodial resilience, etc.” (Mowder et al., 2010; Gibson and Clarbour, 2017).

As noted, further research is needed to explore the basic workings of resilience in context, such as the understanding of the relationship between factors linked to the onset and maintenance of maladjusted behavior and in examining criminal trajectories of those who are offenders (Carr and Vandiver, 2001; Lodewijks et al., 2010; Mowder et al., 2010; Fougere and Daffern, 2011; Gibson and Clarbour, 2017). Although the association between resilience and life condition in the foster and custodial care of children and adolescents was not itself the purpose of the current study, knowing resources and vulnerabilities of such institutional contexts is helpful for providing adequate help and promoting improvement and change. It would require valid measurement by which it is possible to identify and reduce feelings of depression and stress, and also increase support life quality (Mowder et al., 2010; Gibson, 2016; Zuill, 2016; Gibson and Clarbour, 2017). This would improve more informative comparisons across studies and specific policies for purposes of prevention.

This current study also provides important input for assessing resilience among young people in correctional, education, and sociotherapeutic centers (Windle et al., 2011). Such information noted to be lacking in a review of research demanded that such population should be involved in the development of measurement tools. This demand was possible to address, at least in regard to the Polish validation of the RS-14 Scale. “Analyses comparing the strength of this measure among adolescents living in adverse conditions with those who are not can be more accurately conducted and can provide more insight into the validity of this instrument”. Consequently, this current study recruited “higher” and “lower” risk adolescent samples, assumed that all adolescents are not living stress-free lives, and aimed to explore the effect of cumulative stressors within an adolescent resilience framework. “Because adversity is present to a substantial degree across all groups, the simple difference in age alone between the children and adolescents, and between the adolescents and young adults does not really enable reliable discrimination between the proportion of the sample experiencing adversity.” The already established validation of RS-14 for those three populations and especially those adolescents with special needs, externalizing behavioral and juridical maladjustment may open a new trial for further validation of RS-14 with specific populations (Pritzker and Minter, 2014).

Any limitations of these studies are due to the unavailability of the adolescents' actual assessment data on the psychosocial disturbance and maladjustment/offender behavior. In addition, the reasons for their placement in custodial center were not directly analyzed, but rather we assumed formally confirmed adversities and impairments through psych-pedagogical and justice diagnostics, done by experts. The fact of being in socioterapeuthic, educational, or correctional centers can to a degree deliver important, formally proved assessment (psychological, educational and juridical evidence) as can the information of reasons for the delivery to educational care regarding disturbed or maladjusted psychosocial functioning of an adolescent. That is why the present study simply assumes this fact and takes into consideration the additional stressor of being exposed to adverse conditions in the educational or correctional care.

The RS-14's single-factor structure may limit its utility in identifying distinct domains of strength or weakness in resilience particular to an individual and to contextual conditions. Nevertheless, at the same time it gives important insight for individual resources. This appears to be a worthwhile pursuit, particularly in the offender context, as much of the resiliency literature has not examined resilience in youth who offend (Carr and Vandiver, 2001; Lodewijks et al., 2010; Mowder et al., 2010; Fougere and Daffern, 2011; Gibson and Clarbour, 2017). Researchers have supported the opinion that it is personal, individual factors that explain resilience rather than external factors, such as family and community support, that are most important in helping the individual withstand hardship. This may be because people with high resilience also have the personal, individual factors “which are more likely to effectively meet the challenges of their lives, flexibly adapt to the stresses of their lives, and even become successful, healthy, and happy in the future” (Ong et al., 2006; Cohn et al., 2009; Ali et al., 2010; Fletcher and Sarkar, 2013; Liu et al., 2013).

This current study explored and confirmed that the RS-14 could be used to provide a psychometrically validated and consistent measure of individual resilience across all three samples: adolescents, young adults, and those who live with adverse conditions in custodial care (the educational, socioteherapeutic, probation centers). While just few studies with adolescents have validated RS-14, the present study confirms the findings of Pritzker and Minter (2014) regarding the strength of this measure across age groups and suggests its utility not only with both early and middle adolescents and young adults, but also with and within those adolescents belonging to problem group. Despite limitations, the present studies contribute meaningfully to broaden the knowledge about the RS-14 assessment of the resilience, pointing particularly toward the importance of personal resources of an individual in understanding adaptation to adverse life condition and events, especially for adolescents, young adults, and those with emotional and behavioral disturbance and those who are young offenders. That is why the adaptation of RS-14 as a valid brief measure of resilience is useful not only with adolescents, and young adults but also for a cross-group with special needs and maladjustment. The adaptation of RS-14 can be used more consistently across research studies and practice settings not only in Poland. This is obviously an exploratory approach and should be continued in the course of further research.

## Ethics Statement

Ethical approval for the study was granted by the Ethics Committee of the Department of Pedagogy and Psychology at the University Białystok, the Ministry of Justice, and the Centre for Education Development. While consent was sought directly from adult participants, for younger persons consent was also gained from the principals. Within the establishment, contact was made with the manager responsible for the unit in order to determine whether any young people could be invited to take part in the study. We have obtained written informed consent for participation in research from all adult participants and from the parents/legal guardians of all non-adult participants. Participants were informed 1 month in advance about the data collection. Reassurances were made that participants only should participate at their free will and could decline at any time.

## Author Contributions

The contribution of JS and KK was in data collection, conception and design, data analysis, and writing the paper. The contribution of GW was in critical revision for important intellectual content.

### Conflict of Interest Statement

The authors declare that the research was conducted in the absence of any commercial or financial relationships that could be construed as a potential conflict of interest.

## References

[B1] Abiola T. Udofia O. (2011). Psychometric assessment of the Wagnild and Young's resilience scale in Kano, Nigeria. BMC Res. Notes 4:509. 10.1186/1756-0500-4-509. 22112503 PMC3261834

[B2] Abolghasemi A. Varaniyab S. T. (2010). Resilience and perceived stress: predictors of life satisfaction in the students of success and failure. Proc. Soc. Behav. Sci. 5, 748–752. 10.1016/j.sbspro.2010.07.178

[B3] Ahern N. R. Kiehl E. M. Sole M. L. Byers J. (2006). A review of instruments measuring resilience. Issues Compr. Pediatr. Nurs. 29, 103–125. 10.1080/01460860600677643 16772239

[B4] Aiena B. J. Baczwaski B. J. Schulenberg S. E. Buchanan E. M. (2015). Measuring resilience with the RS-14: a tale of two samples. J. Pers. Assess. 97, 291–300. 10.1080/00223891.2014.951445 25257682

[B5] Ali M. M. Dwyer D. S. Vanner E. A. Lopez A. (2010). Adolescent propensity to engage in health risky behaviors: the role of individual resilience. Int. J. Environ. Res. Public Health 7, 2161–2176. 10.3390/ijerph7052161 20623017 PMC2898042

[B6] Aroian K. J. Schappler-Morris N. Neary S. Spitzer A. Tran T. V. (1997). Psychometric evaluation of the russian language version of the resilience scale. J. Nurs. Meas. 5, 151–164. 10.1891/1061-3749.5.2.151 9538587

[B7] Artuch-Garde R. González-Torres M. D. C. de la Fuente J. Vera M. M. Fernández-Cabezas M. López-García M.. (2017). Relationship between resilience and self-regulation: a study of spanish youth at risk of social exclusion. Front. Psychol. 8:612. 10.3389/fpsyg.2017.00612 28473792 PMC5397523

[B8] Avanci J. Q. Assis S. G. Oliveira R. V. C. Ferreira R. M. Pesce R. P. (2007). Associated factors with mental health problems in adolescents. Psicologia: Teoria e Pesquisa. 23, 287–294. 10.1590/S0102-37722007000300007

[B9] Beasley M. Thompson T. Davidson J. (2003). Resilience in response to life stress: the effects of coping style and cognitive hardiness. Pers. Individ. Dif. 34, 77–95. 10.1016/S0191-8869(02)00027-2

[B10] Block J. Kremen A. M. (1996). IQ and ego-resiliency: conceptual and empirical connections and separateness. J. Pers. Soc. Psychol. 70, 349–361. 10.1037/0022-3514.70.2.349 8636887

[B11] Bonanno G. A. Kennedy P. Galatzer-Levy I. R. Lude P. Elfström M. L. (2012). Trajectories of resilience, depression, and anxiety following spinal cord injury. Rehabil. Psychol. 57, 236–247. 10.1037/a0029256 22946611

[B12] Borum R. Bartel P. Forth A. (2002). SAVRY: Manual for the Structured Assessment of Violence Risk in Youth. Tampa, FL: Florida Mental Health Institute, University of South Florida.

[B13] Brooks S. J. Krulewicz S. P. Kutcher S. (2003). The kutcher adolescent depression scale: assessment of its evaluative properties over the course of an 8-week pediatric pharmacotherapy trial. J. Child Adolesc. Psychopharmacol. 13, 337–349. 10.1089/104454603322572679 14642022

[B14] Bruce E. Naccarato T. Hopson L. Morrelli K. (2010). Providing a sound educational framework for foster youth: a proposed research agenda. J. Public Child Welf. 4, 219–240. 10.1080/15548731003799688

[B15] Burt K. B. Paysnick A. A. (2012). Resilience in the transition to adulthood. Dev. Psychopathol. 24, 493–505. 10.1017/S0954579412000119 22559126

[B16] Callegari C. Bertù L. Lucano M. Ielmini M. Braggio E. Vender S. (2016). Reliability and validity of the Italian version of the 14-item Resilience Scale. Psychol. Res. Behav. Manag. 9, 277–284. 10.2147/PRBM.S115657 27757055 PMC5055039

[B17] Campbell-Sills L. Cohan S. L. Stein M. B. (2006). Relationship of resilience to personality, coping, and psychiatric symptoms in young adults. Behav. Res. Ther. 44, 585–599. 10.1016/j.brat.2005.05.001 15998508

[B18] Campbell-Sills L. Stein M. B. (2007). Psychometric analysis and refinement of the connor–davidson resilience scale (CD-RISC): validation of a 10-item measure of resilience. J. Trauma. Stress 20, 1019–1028. 10.1002/jts.20271 18157881

[B19] Carr M. B. Vandiver T. A. (2001). Risk and protective factors among youth offenders. Adolescence, 36, 409–426. 11817624

[B20] Castillo J. A. G. Dias P. C. (2009). Auto-regulação, resiliência e consumo de substâncias na adolescência: contributos da adaptação do questionário reduzido de auto-regulação. Psicologia Saúde amp Doenças 10, 205–216.

[B21] Catalano D. Chan F. Wilson L. Chiu C. Y. Muller V. R. (2011). The buffering effect of resilience on depression among individuals with spinal cord injury: a structural equation model. Rehabil. Psychol. 56, 200–211. 10.1037/a0024571 21843016

[B22] Catalano R. F. Berglund M. L. Ryan J. A. Lonczak H. S. Hawkins J. D. (2004). Positive youth development in the United States: research findings on evaluations of positive youth development programs. Ann. Am. Acad. Pol. Soc. Sci. 591, 98–124. 10.1177/0002716203260102

[B23] Cicchetti D. V. (1994). Guidelines, criteria, and rules of thumb for evaluating normed and standardized assessment instruments in psychology. Psychol. Assess. 6, 284–290. 10.1037/1040-3590.6.4.284

[B24] Cohen S. Kamarck T. Mermelstein R. (1983). A global measure of perceived stress. J. Health Soc. Behav. 24, 385–396. 10.2307/2136404 6668417

[B25] Cohn M. A. Fredrickson B. L. Brown S. L. Mikels J. A. Conway A. M. (2009). Happiness unpacked: positive emotions increase life satisfaction by building resilience. Emotion 9, 361–368. 10.1037/a0015952 19485613 PMC3126102

[B26] Coleman J. Hagell A. (2007a). Adolescence, risk and resilience: Against the odds, (Vol. 3). New York, NY: John Wiley & Sons.

[B27] Coleman J. Hagell A. (2007b). The nature of risk and resilience in adolescence, in Adolescence, Risk and Resilience: Against the Odds, eds Coleman J. Hagell A. (West Sussex: John Wiley & Sons), 1–16.

[B28] Damásio B. F. Borsa J. C. da Silva J. P. (2011). 14-item resilience scale (rs-14): psychometric properties of the brazilian version. J. Nurs. Meas. 19, 131–145. 10.1891/1061-3749.19.3.131 22372090

[B29] Diener E. Emmons R. A. Larsen R. J. Griffin S. (1985). The satisfaction with life scale. J. Pers. Assess. 49, 71–75. 10.1207/s15327752jpa4901_13 16367493

[B30] Diener E. Suh E. M. Lucas R. E. Smith H. L. (1999). Subjective well-being: three decades of progress. Psychol. Bull. 125:276 10.1037/0033-2909.125.2.276

[B31] Donnellan W. J. Bennett K. M. Soulsby L. K. (2015). What are the factors that facilitate or hinder resilience in older spousal dementia carers? A qualitative study. Aging Ment. Health 19, 932–939. 10.1080/13607863.2014.977771 25410637

[B32] Earvolino-Ramirez M. (2007). Resilience: a concept analysis. Nurs. Forum 42, 73–82. 10.1111/j.1744-6198.2007.00070.x 17474940

[B33] Erkut S. (2010). Developing multiple language versions of instruments for intercultural research. Child Dev. Perspect. 4, 19–24. 10.1111/j.1750-8606.2009.00111.x 21423824 PMC3060794

[B34] Feder A. Nestler E. J. Westphal M. Charney D. S. (2010). Psychobiological mechanisms of resilience to stress, in Handbook of Adult Resilience, eds Reich J. W. Zautra A. J. Hall J. S. (New York, NY: The Guilford Press), 35–54.

[B35] Fergus S. Zimmerman M. A. (2005). Adolescent resilience: a framework for understanding healthy development in the face of risk. Annu. Rev. Public Health 26, 399–419. 10.1146/annurev.publhealth.26.021304.144357 15760295

[B36] Fletcher D. Sarkar M. (2012). A grounded theory of psychological resilience in olympic champions. Psychol. Sport Exerc. 13, 669–678. 10.1016/j.psychsport.2012.04.007

[B37] Fletcher D. Sarkar M. (2013). Psychological resilience. Europ. Psychol. 18, 12–23. 10.1027/1016-9040/a000124

[B38] Folkman S. Moskowitz J. T. (2004). Coping: pitfalls and promise. Annu. Rev. Psychol. 55, 745–774. 10.1146/annurev.psych.55.090902.141456 14744233

[B39] Fougere A. Daffern M. (2011). Resilience in young offenders. Int. J. Forensic Ment. Health 10, 244–253. 10.1080/14999013.2011.598602

[B40] Fredrickson B. L. Tugade M. M. Waugh C. E. Larkin G. R. (2003). What good are positive emotions in crisis? a prospective study of resilience and emotions following the terrorist attacks on the United States on September 11th, 2001. J. Personal. Soc. Psychol. 84, 365–376. 10.1037/0022-3514.84.2.365 PMC2755263 12585810

[B41] Gardner T. W. Dishion T. J. Connell A. M. (2008). Adolescent self-regulation as resilience: resistance to antisocial behavior within the deviant peer context. J. Abnorm. Child Psychol. 36, 273–284. 10.1007/s10802-007-9176-6 17899361

[B42] Gartland D. Bond L. Olsson C. A. Buzwell S. Sawyer S. M. (2011). Development of a multi-dimensional measure of resilience in adolescents: the adolescent resilience questionnaire. BMC Med. Res. Methodol. 11:134. 10.1186/1471-2288-11-134 21970409 PMC3204306

[B43] Gibson R. A. (2016). A Psychometric Study of Resilience and Custodial Adjustment Among Young People in Custody. Ph.D thesis. University of York.

[B44] Gibson R. A. Clarbour J. (2017). Factorial structure of the Resiliency Scale for Children and Adolescents (RSCA) among incarcerated male adolescent offenders. J. For. Pract. 19, 23–36. 10.1108/JFP-08-2015-0043

[B45] Grotberg E. H. (1995). A Guide to Promoting Resilience in Children: Strengthening the Human Spirit. The Hague: Bernard van leer foundation Available online at: http://www.bibalex.org/Search4Dev/files/283337/115519.pdf

[B46] Hall C. W. Webster R. E. (2007a). Multiple stressors and adjustment among adult children of alcoholics. Addict. Res. Theor. 15, 425–434. 10.1080/16066350701261865

[B47] Hall C. W. Webster R. E. (2007b). Risk factors among adult children of alcoholics. Int. J. Behav. Consult. Ther. 3, 494–511. 10.1037/h0100819

[B48] Harkness J. A. Villar A. Edwards B. (2010). Translation, adaptation, and design, in Wiley Series in Survey Methodology. Survey Methods in Multinational, Multiregional, and Multicultural Contexts, eds Harkness J. A. Braun M. Edwards B. Johnson T. P. Lyberg L. Mohler P. P. Smith T. W. (Hoboken, NJ: John Wiley & Sons Inc.), 117–140.

[B49] Harvey J. Delfabbro P. H. (2004). Psychological resilience in disadvantaged youth: a critical overview. Aust. Psychol. 39, 3–13. 10.1080/00050060410001660281

[B50] Heilemann M. V. Lee K. Kury F. S. (2003). Psychometric properties of the spanish version of the resilience scale. J. Nurs. Meas. 11, 61–72. 10.1891/jnum.11.1.61.52067 15132012

[B51] Hjemdal O. (2007). Measuring protective factors: the development of two resilience scales in Norway. Child Adolesc. Psychiatric Clin. 16, 303–321. 10.1016/j.chc.2006.12.003 17349510

[B52] Hjemdal O. Vogel P. A. Solem S. Hagen K. Stiles T. C. (2011). The relationship between resilience and levels of anxiety, depression, and obsessive–compulsive symptoms in adolescents. Clin. Psychol. Psychother. 18, 314–321. 10.1002/cpp.719 20806419

[B53] Hu L. Bentler P. M. (1999). Cutoff criteria for fit indexes in covariance structure analysis: conventional criteria versus new alternatives. Struct. Equ. Model. Multidisciplinary J. 6, 1–55. 10.1080/10705519909540118

[B54] Jaffee S. R. Gallop R. (2007). Social, emotional, and academic competence among children who have had contact with child protective services: prevalence and stability estimates. J. Am. Acad. Child Adolescent Psychiatry 46, 757–765. 10.1097/chi.0b013e318040b247 17513988 PMC3703530

[B55] Jain S. Cohen A. K. (2013). Fostering resilience among urban youth exposed to violence: a promising area for interdisciplinary research and practice. Health Edu. Behav. 40, 651–662. 10.1177/1090198113492761 23818463

[B56] Jørgensen I. E. Seedat S. (2008). Factor structure of the Connor-Davidson resilience scale in South African adolescents. Int. J. Adolesc. Med. Health 20, 3–32. 10.1515/IJAMH.2008.20.1.23 18540281

[B57] Juczynski Z. (1999). Narzedzia pomiaru w psychologii zdrowia. Przeglad Psychologiczny 42, 43–56.

[B58] Juczynski Z. Oginska-Bulik N. (2012). Narzedzia Pomiaru Stresu i Radzenia Sobie ze Stresem. Pracownia Testów Psychologicznych Polskiego Towarzystw Psychologicznego. Avaliable online at: http://scholar.google.com/scholar?cluster=6997986605580809144&hl=en&oi=scholarr

[B59] Kaczmarek Ł. D. (2011). Kwestionariusz Sprezystości Psychicznej – Polska Adaptacja Ego Resiliency Scale. Adaptation and Validation of Ego Resiliency Scale into Polish. Avilable online at: https://www.researchgate.net/publication/216100514_Kwestionariusz_Sprezystosci_Psychicznej_-_polska_adaptacja_Ego_Resiliency_Scale_Adaptation_and_Validation_of_Ego_Resiliency_Scale_into_Polish (Accessed March 3, 2017).

[B60] Kwon H. J. Kwon S. J. (2014). Korean version of the 14-Item resilience scale (RS-14) for university students: a validity and reliability study. J. Korean Acad. Psychiatric Mental Health Nurs. 23, 226–232. 10.12934/jkpmhn.2014.23.4.226

[B61] Liu Y. Wang Z. Lü W. (2013). Resilience and affect balance as mediators between trait emotional intelligence and life satisfaction. Pers. Individ. Dif. 54, 850–855. 10.1016/j.paid.2012.12.010

[B62] Lodewijks H. P. de Ruiter C. Doreleijers T. A. (2010). The impact of protective factors in desistance from violent reoffending: a study in three samples of adolescent offenders. J. Interpers. Violence 25, 568–587. 10.1177/0886260509334403 19584407

[B63] Losoi H. Turunen S. Wäljas M. Helminen M. Öhman J. Julkunen J. (2013). Psychometric properties of the finnish version of the resilience scale and its short version. Psychol. Commu. Health 2, 1–10. 10.5964/pch.v2i1.40

[B64] Lundman B. Strandberg G. Eisemann M. Gustafson Y. Brulin C. (2007). Psychometric properties of the swedish version of the resilience scale. Scand. J. Caring Sci. 21, 229–237. 10.1111/j.1471-6712.2007.00461.x 17559442

[B65] Lutha S. S. Cicchetti D. (2000). The construct of resilience: implications for interventions and social policies. Dev. Psychopathol. 12, 857–885. 10.1017/S0954579400004156 11202047 PMC1903337

[B66] Luthar S. S. (2006). Resilience in Development: A Synthesis of Research Across Five Decades. Avalable online at: http://psycnet.apa.org/psycinfo/2006-03609-020

[B67] Luthar S. S. Cicchetti D. Becker B. (2000). Research on resilience: response to commentaries. Child Develop. 71, 573–575. 10.1111/1467-8624.00168 PMC1885202 10953923

[B68] Madewell A. N. Ponce-Garcia E. (2016). Assessing resilience in emerging adulthood: the resilience scale (RS), connor–davidson resilience scale (CD-RISC), and scale of protective factors (SPF). Pers. Individ. Dif. 97, 249–255. 10.1016/j.paid.2016.03.036

[B69] Mancini A. D. Bonanno G. A. (2009). Predictors and parameters of resilience to loss: toward an individual differences model. J. Pers. 77, 1805–1832. 10.1111/j.1467-6494.2009.00601.x 19807863 PMC4224188

[B70] Masten A. S. (2011). Resilience in children threatened by extreme adversity: frameworks for research, practice, and translational synergy. Dev. Psychopathol. 23, 493–506. 10.1017/S0954579411000198 23786691

[B71] Masten A. S. Narayan A. J. (2012). Child development in the context of disaster, war, and terrorism: pathways of risk and resilience. Annu. Rev. Psychol. 63, 227–257. 10.1146/annurev-psych-120710-100356 21943168 PMC5858878

[B72] Mastropieri M. A. Scruggs T. E. (2010). The Inclusive Classroom: Strategies for Effective Differentiated Instruction. River, NJ: Merrill Upper Saddle.

[B73] Mojs E. Bartkowska W. Kaczmarek Lukasz D. Ziarko M. Bujacz A. Warchol-Biedermann K. (2015). Wlaściwości psychometryczne polskiej wersji skróconej Skali Depresji Kutchera dla Mlodziezy (Kutcher Adolescent Depression Scale)–pomiar depresji w grupie studentów. Psychiatr. Pol. 49, 135–144. 10.12740/PP/22934 25844416

[B74] Montpetit M. A. Bergeman C. S. Deboeck P. R. Tiberio S. S. Boker S. M. (2010). Resilience-as-process: negative affect, stress, and coupled dynamical systems. Psychol. Aging 25, 631–640. 10.1037/a0019268 20853969

[B75] Mowder M. H. Cummings J. A. McKinney R. (2010). Resiliency scales for children and adolescents: profiles of juvenile offenders. J. Psychoeduc. Assess. 28, 326–337. 10.1177/0734282910366838

[B76] Nishi D. Uehara R. Kondo M. Matsuoka Y. (2010). Reliability and validity of the Japanese version of the resilience scale and its short version. BMC Res. Notes 3:310. 10.1186/1756-0500-3-310 21083895 PMC2993730

[B77] Ntountoulaki E. Paika V. Kotsis K. Papaioannou D. Andreoulakis E. Fountoulakis K. N. (2017). The Greek version of the resilience scale (rs-14): psychometric properties in three samples and associations with mental illness, Suicidality, and Quality of Life. J. Psychol. Clin. Psychiatry 7:00450 10.15406/jpcpy.2017.07.00450 PMC5314716 28239407

[B78] Nygren B. Aléx L. Jonsén E. Gustafson Y. Norberg A. Lundman B. (2005). Resilience, sense of coherence, purpose in life and self-transcendence in relation to perceived physical and mental health among the oldest old. Aging Mental Health 9, 354–362. 10.1080/1360500114415 16019292

[B79] Oginska-Bulik N. Juczynski Z. (2011). Prezność u dzieci i młodziezy: charakterystyka i pomiar – polska skala SPP−18. Polskie Forum Psychol. 1, 7–28.

[B80] Oginska-Bulik N. Kobylarczyk M. (2016). The mediating role of resiliency in the relationship between temperament and posttraumatic growth. J. Loss Trauma 22, 1–10. 10.1080/15325024.2016.1159115

[B81] Oliveira A. Matos A. P. do Rosário Pinheiro M. Oliveira S. (2015). Confirmatory factor analysis of the resilience scale short form in a portuguese adolescent sample. Proc. Soc. Behav. Sci. 165, 260–266. 10.1016/j.sbspro.2014.12.630

[B82] Ong A. D. Bergeman C. S. Bisconti T. L. Wallace K. A. (2006). Psychological resilience, positive emotions, and successful adaptation to stress in later life. J. Pers. Soc. Psychol. 91, 730–749. 10.1037/0022-3514.91.4.730 17014296

[B83] Pangallo A. Zibarras L. Lewis R. Flaxman P. (2015). Resilience through the lens of interactionism: a systematic review. Psychol. Assess. 27, 1–20. 10.1037/pas0000024 25222438

[B84] Patry D. Ford R. (2016). Measuring Resilience as an Education Outcome. Higher Education Quality Council of Ontario.

[B85] Pecora P. J. (2012). Maximizing educational achievement of youth in foster care and alumni: factors associated with success. Child. Youth Serv. Rev. 34, 1121–1129. 10.1016/j.childyouth.2012.01.044

[B86] Portzky M. Wagnild G. De Bacquer D. Audenaert K. (2010). Psychometric evaluation of the dutch resilience scale RS-nl on 3265 healthy participants: a confirmation of the association between age and resilience found with the Swedish version. Scand. J. Caring Sci. 24, 86–92. 10.1111/j.1471-6712.2010.00841.x 21070312

[B87] Prince-Embury S. (2008). The resiliency scales for children and adolescents, psychological symptoms, and clinical status in adolescents. Can. J. School Psychol. 23, 41–56. 10.1177/0829573508316592

[B88] Prince-Embury S. Courville T. (2008). Measurement invariance of the resiliency scales for children and adolescents with respect to sex and age cohorts. Can. J. School Psychol. 23, 26–40. 10.1177/0829573508316590

[B89] Pritzker S. Minter A. (2014). Measuring adolescent resilience: an examination of the cross-ethnic validity of the RS-14. Child. Youth Serv. Rev. 44, 328–333. 10.1016/j.childyouth.2014.06.022

[B90] Quinn M. M. Rutherford R. B. Leone P. E. Osher D. M. Poirier J. M. (2005). Youth with disabilities in juvenile corrections: a national survey. Except. Child. 71, 339–345. 10.1177/001440290507100308

[B91] Rubin K. H. Bukowski W. M. Parker J. G. (1998). Peer interactions, relationships, and groups, in Handbook of Child Psychology, Social, Emotional, and Personality Development, Vol. 3, 5th Edn, ed Eisenberg N. (New York, NY: Wiley).

[B92] Rutter M. (1999). Resilience concepts and findings: implications for family therapy. J. Fam. Ther. 21, 119–144. 10.1111/1467-6427.00108

[B93] Rutter M. (2012). Resilience as a dynamic concept. Dev. Psychopathol. 24, 335–344. 10.1017/S0954579412000028 22559117

[B94] Sagone E. De Caroli M. E. (2013). Relationships between resilience, self-efficacy, and thinking styles in Italian middle adolescents. Proc. Soc. Behav. Sci. 92, 838–845. 10.1016/j.sbspro.2013.08.763

[B95] Sagone E. De Caroli M. E. (2014). Relationships between psychological well-being and resilience in middle and late adolescents. Proc. Soc. Behav. Sci. 141, 881–887. 10.1016/j.sbspro.2014.05.154

[B96] Salazar-Pousada D. Arroyo D. Hidalgo L. Pérez-López F. R. Chedraui P. (2010). Depressive symptoms and resilience among pregnant adolescents: a case-control study. Obstet. Gynecol. Int. 2010:952493. 10.1155/2010/952493 21461335 PMC3065659

[B97] Sánchez-Teruel D. María Auxiliadora R.-B. (2016). 14-Item Resilience Scale (RS-14): Psychometric Properties of the Spanish Version. Revista Iberoamericana de Diagnóstico y Evaluación Psicológica, Vol. 2, N° 40, 2015, 103–113.

[B98] Scali J. Gandubert C. Ritchie K. Soulier M. Ancelin M. L. Chaudieu I. (2012). Measuring resilience in adult women using the 10-items Connor-Davidson Resilience Scale (CD-RISC). Role of trauma exposure anxiety disorders. PLoS ONE 7:e39879. 10.1371/journal.pone.0039879 22768152 PMC3387225

[B99] Schumacher J. Leppert K. Gunzelmann T. Strauß B. Brähler E. (2005). Die resilienzskala–ein fragebogen zur erfassung der psychischen widerstandsfähigkeit als personmerkmal. Z. Klin. Psychol. Psychiatr. Psychother. 53, 16–39.

[B100] Schwarzer R. Warner L. M. (2013). Perceived self-efficacy and its relationship to resilience, in The Springer Series on Human Exceptionality. Resilience in Children, Adolescents, and Adults: Translating Research Into Practice, eds Prince-Embury S. Saklofske D. H. (New York, NY: Springer Science + Business Media), 139–150. 10.1007/978-1-4614-4939-3_10

[B101] Scruggs T. E. Mastropieri M. A. Berkeley S. Graetz J. E. (2010). Do special education interventions improve learning of secondary content? a meta-analysis. Remedial Spec. Edu. 31, 437–449. 10.1177/0741932508327465

[B102] Seita J. R. Brown W. K. (2010). Reclaiming Children and Youth, Vol 18, Bloomington, IN: Publisher Starr Global Learning Network, Nr 4 (Winter 2010)55–58.

[B103] Sharaf A. Y. Thompson E. A. Walsh E. (2009). Protective effects of self-esteem and family support on suicide risk behaviors among at-risk adolescents. J. Child Adolescent Psychiatr. Nurs. 22, 160–168. 10.1111/j.1744-6171.2009.00194.x 19702970

[B104] Smith-Osborne A. Whitehill Bolton K. (2013). Assessing resilience: a review of measures across the life course. J. Evid. Based Soc. Work 10, 111–126. 10.1080/15433714.2011.597305 23581805

[B105] Southwick S. M. Vythilingam M. Charney D. S. (2005). The psychobiology of depression and resilience to stress: implications for prevention and treatment. Annu. Rev. Clin. Psychol. 1, 255–291. 10.1146/annurev.clinpsy.1.102803.143948 17716089

[B106] Steiger J. H. (1990). Structural model evaluation and modification: an interval estimation approach. Multi. Behav. Res. 25, 173–180. 10.1207/s15327906mbr2502_4 26794479

[B107] Surtees P. G. Wainwright N. W. Khaw K. T. (2006). Resilience, misfortune, and mortality: evidence that sense of coherence is a marker of social stress adaptive capacity. J. Psychosom. Res. 61, 221–227. 10.1016/j.jpsychores.2006.02.014 16880025

[B108] Taku K. (2014). Relationships among perceived psychological growth, resilience and burnout in physicians. Pers. Individ. Dif. 59, 120–123. 10.1016/j.paid.2013.11.003

[B109] Tempski P. Santos I. S. Mayer F. B. Enns S. C. Perotta B. Paro H. B.. (2015). Relationship among medical student resilience, educational environment and quality of life. PLoS ONE 10:e0131535. 10.1371/journal.pone.0131535 26121357 PMC4486187

[B110] Tian J. Hong J. S. (2013). Validation of the Chinese version of the resilience scale and its cutoff score for detecting low resilience in Chinese cancer patients. Support. Care Cancer 21, 1497–1502. 10.1007/s00520-012-1699-x 23274927

[B111] Tucker L. R. Lewis C. (1973). A reliability coefficient for maximum likelihood factor analysis. Psychometrika 38, 1–10. 10.1007/BF02291170

[B112] Tugade M. M. Fredrickson B. L. (2004). Resilient individuals use positive emotions to bounce back from negative emotional experiences. J. Pers. Soc. Psychol. 86, 320–333. 10.1037/0022-3514.86.2.320 14769087 PMC3132556

[B113] Uchnast Z. (1998). Prezność osobowa a egzystencjalne wymiary wartościowania. Roczniki Psychol. 1, 7–27.

[B114] Ungar M. Liebenberg L. (2009). Cross-cultural consultation leading to the development of a valid measure of youth resilience: the international resilience project. Stud. Psychol. 51, 259–268.

[B115] Ungar M. Liebenberg L. (2011). Assessing resilience across cultures using mixed methods: construction of the child and youth resilience measure. J. Mixed Methods Res. 5, 126–149. 10.1177/1558689811400607

[B116] Veselska Z. Geckova A. M. Orosova O. Gajdosova B. van Dijk J. P. Reijneveld S. A. (2009). Self-esteem and resilience: the connection with risky behavior among adolescents. Addict. Behav. 34, 287–291. 10.1016/j.addbeh.2008.11.005 19056183

[B117] Wagnild G. (2009a). A review of the resilience scale. J. Nurs. Meas. 17, 105–113. 10.1891/1061-3749.17.2.105 19711709

[B118] Wagnild G. (2009b). The Resilience Scale User's Guide for the US Englishversion of the Resilience Scale and the 14-Item Resilience Scale (RS-14). Worden, MT: Resilience Center.

[B119] Wagnild G. (2014). True resilience: building a life of strength, courage, and meaning: an interactive guide, in Cape House Books.

[B120] Wagnild G. M. Young H. M. (1993). Development and psychometric evaluation of the Resilience Scale. J. Nurs. Meas. 1, 165–178. 7850498

[B121] Wiles J. L. Wild K. Kerse N. Allen R. E. (2012). Resilience from the point of view of older people:‘There’s still life beyond a funny knee.' Soc. Sci. Med. 74, 416–424. 10.1016/j.socscimed.2011.11.005 22204841

[B122] Windle G. Bennett K. M. Noyes J. (2011). A methodological review of resilience measurement scales. Health Qual. Life Outcomes 9:8. 10.1186/1477-7525-9-8 21294858 PMC3042897

[B123] Wong P. T. P. Wong L. C. J. (2012). A meaning-centered approach to building youth resilience, in Personality and Clinical Psychology Series. The Human Quest for Meaning: Theories, Research, and Applications, ed Wong P. T. P. (New York, NY: Routledge/Taylor & Francis Group), 585–617.

[B124] Wright M. O. Masten A. S. (2005). Resilience processes in development, in Handbook of Resilience in Children (Springer), 17–37. Available online at: http://link.springer.com/chapter/10.1007/0-306-48572-9_2 10.1007/0-306-48572-9_2

[B125] Wyche K. F. Pfefferbaum R. L. Pfefferbaum B. Norris F. H. Wisnieski D. Younger H. (2011). Exploring community resilience in workforce communities of first responders serving Katrina survivors. Am. J. Orthopsychiatry 81, 18–30. 10.1111/j.1939-0025.2010.01068.x 21219272

[B126] Yang Y. Li M.-H. Xia Y. (2012). Measurement invariance of the resilience scale. Int. J. Edu. Psychol. Assess. 11, 1–19.

[B127] Yarcheski A. Mahon N. E. (1999). The moderator-mediator role of social support in early adolescents. West. J. Nurs. Res. 21, 685–698. 10.1177/01939459922044126 11512187

[B128] Ying L. Wu X. Lin C. Jiang L. (2014). Traumatic severity and trait resilience as predictors of posttraumatic stress disorder and depressive symptoms among adolescent survivors of the Wenchuan earthquake. PLoS ONE 9:e89401. 10.1371/journal.pone.0089401 24586751 PMC3935868

[B129] Zuill Z. D. (2016). The Relationship Between Resilience and Academic Success Among Bermuda Foster Care Adolescents. Ph.D. thesis, Walden University.

